# The Thoraflex hybrid approach using a zone 0 proximal landing site for first-stage elective treatment of a thoracoabdominal aneurysm

**DOI:** 10.1093/jscr/rjad692

**Published:** 2023-12-30

**Authors:** Ramanish Ravishankar, Sanjeet Avtaar Singh, Vincenzo Giordano

**Affiliations:** Faculty of Public Health, London School of Hygiene and Tropical Medicine, Keppel Street, London, WC1E 7HT, United Kingdom; Department of Cardiothoracic Surgery, Golden Jubilee Hospital, Agamemnon St, Clydebank, G81 4DY, United Kingdom; Department of Cardiothoracic Surgery, Royal Infirmary of Edinburgh, 51 Little France Cres, Edinburgh, EH16 4SA, United Kingdom

**Keywords:** cardiac surgery, aortic surgery, frozen elephant trunk, zone 0, thoraco-abdominal aneurysm

## Abstract

A 67-year-old woman was referred to the cardiothoracic outpatient clinic with a long-standing asymptomatic type 2 thoracoabdominal aneurysm. Her CT aorta showed extensive disease in the distal arch with no safe landing zone for total endovascular aneurysm repair (TEVAR). An acute bend preceding the descending aorta also made using a conventional elephant trunk challenging. A multi-disciplinary team decision was made to perform an aortic arch replacement using a frozen elephant trunk at zone 0. Utilizing a zone 0 approach in an elective case can result in quicker organ perfusion and successful TEVAR if necessary.

## Introduction

The Frozen Elephant Trunk (FET) has been incorporated into routine practice over the past decade with ongoing debates regarding FET versus a conventional elephant trunk (CET) [1]. Aside from a Dacron trunk as per the CET, the FET employs a stented distal extension and supra-aortic arch extensions, as seen in the Thoraflex Hybrid Device [2]. Indications for FET have been described in the European Association of Cardio-thoracic Surgery Vascular Domain as extensive aortic tearing distally [2]. This case presents a 67-year-old female who had a two-staged approach for an extensive type 2 thoracoabdominal aneurysm (TAA) starting from zone 0 proximally. After discussions with the vascular surgeons, we performed a staged procedure of a FET followed by subsequent Total Endovascular Aneurysm Repair (TEVAR).

Current literature has established the use of FET in the treatment of Type 2 thoracoabdominal aneurysms [2]. Similarly, the zone 0 approach has also been utilized in the context of aortic dissection [3]. However, in this case, the patient underwent zone 0 anastomosis for an elective TAA repair which is rare in surgical practice.

## Case summary

**Figure 1:**
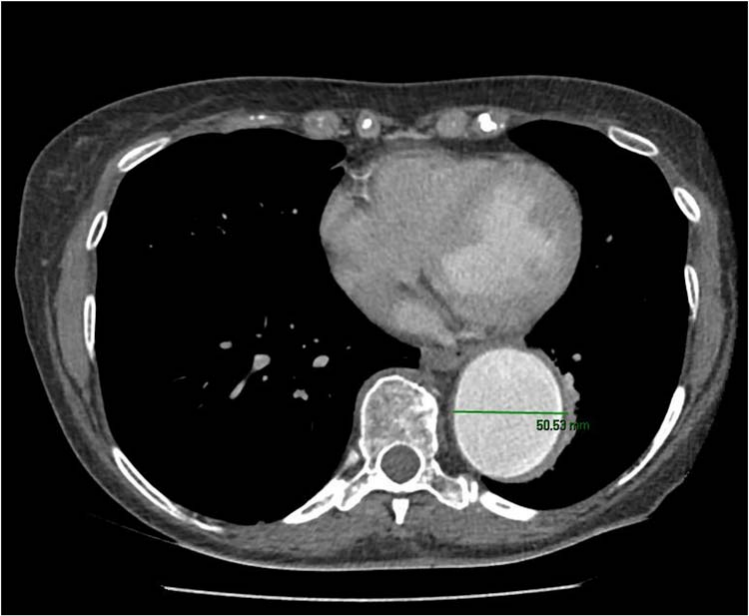
CT scan showing diameter of descending aorta measuring 50 mm.

A 67-year-old female with a history from renal impairment and chronic obstructive pulmonary disease (COPD) as an ex-smoker, presented with a known type 2 TAA. A CT aorta showed an aneurysm starting from the left subclavian artery to the aorto-iliac bifurcation. This measured 52 mm in diameter distally near the bifurcation and 50 mm at the descending aorta. Similarly, the distal aortic arch was dilated at 35–40 mm, and the distal ascending aorta was 45 mm and calcified (Fig. 1).

Initial multi-disciplinary team discussion ruled out TEVAR as there was no suitable landing zone and CT imaging identified an acute bend following the distal arch just before the descending aorta (zone 3 to zone 4). Furthermore, due to this patient’s COPD, an open thoraco-abdominal repair via thoracotomy was considered high risk for post-operative respiratory compromise. Hence, an alternative proposal was adopted with a two-stage operation. The first stage would be undertaken with an FET to zone 0 to provide a safe landing zone for thoraco-abdominal repair. The latter stage would be undertaken as TEVAR or open repair.

**Figure 2:**
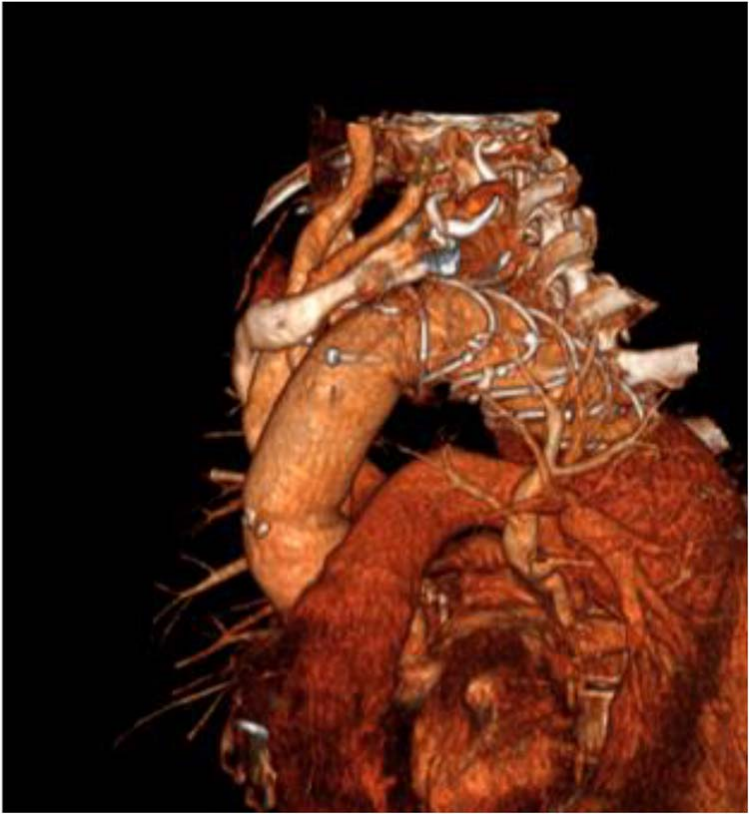
3D reconstructed CT aorta image showing successful FET implantation at zone 0.

**Figure 3:**
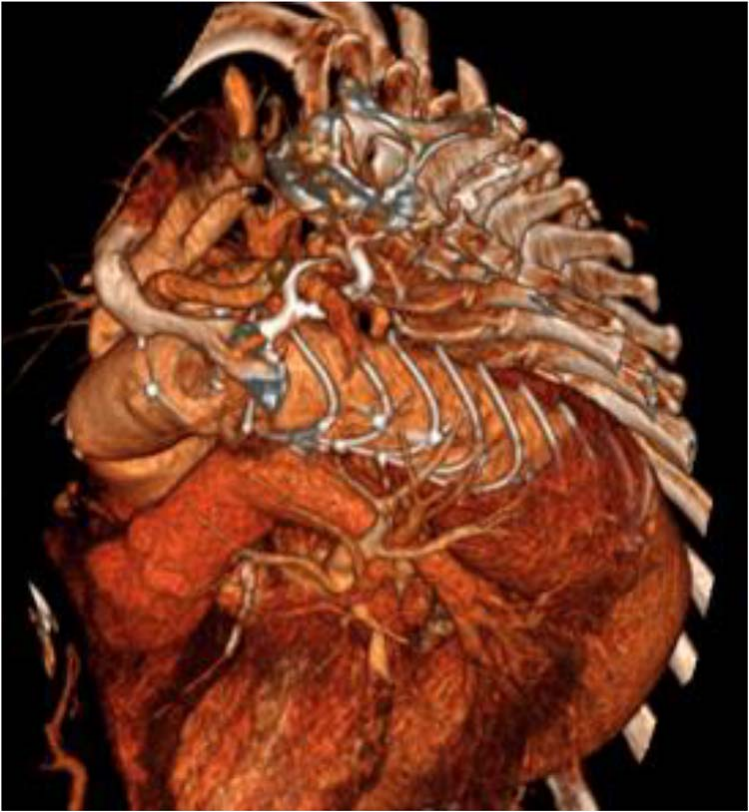
3D reconstructed CT aorta image showing successful FET implantation at zone 0.

**Figure 4:**
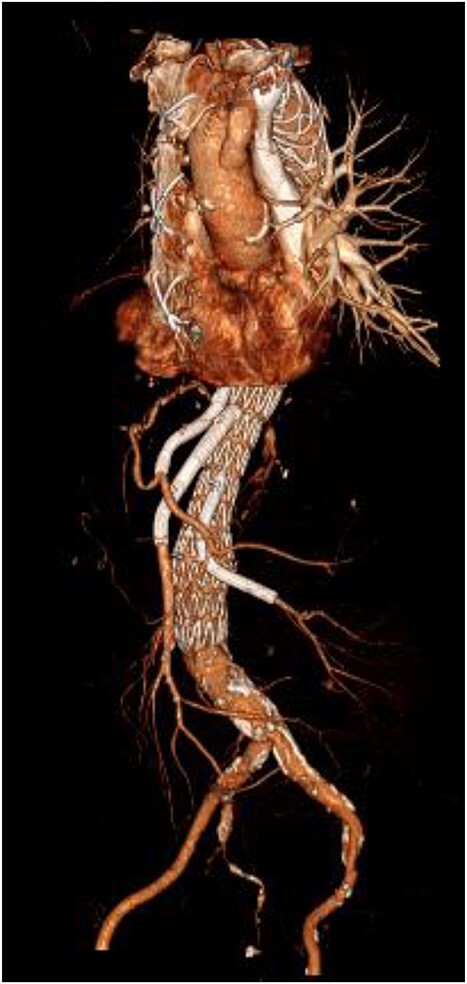
3D reconstructed CT thoracic aorta image showing successful FET implantation and TEVAR.

An 8 mm Gelweave graft was anastomosed to the left subclavian artery. Standard cannulation technique was used with the distal aorta and the right atrium. The distal aorta was sized and a 30/32 mm (15 cm length) Thoraflex graft was anastomosed with 4–0 prolene and reinforced with 1.5 cm Teflon. The supra-aortic vessels were reimplanted separately; deep hypothermia (20°C) was utilized for neuroprotection with antegrade cerebral perfusion and near infrared spectroscopy monitoring. Proximal aorta anastomosis followed patient rewarming. Her post-op CT aorta showed adequate perfusion and FET placement. A 3D reconstruction is shown in Figs 2 and 3. She was discharged on Day 10 and successful TEVAR was undertaken 8 months later (Fig. 4). Follow-up 24 months later showed no evidence of endoleak, stent migration or fracture.

## Discussion

This is an innovative case of utilizing the Thoraflex Hybrid device in the repair of a type 2 thoraco-abdominal aneurysm as the first part of a two-stage process. There were several reasons why zone 0 was used. Firstly, the acute bend in the aorta described on CT meant utilizing CET could lead to subsequent kinking of the Dacron graft. The stented portion of the FET, placed proximally, would help circumvent this.

The contra-indication for thoracotomy leading to open repair was a propagating factor for favouring future TEVAR. Although a CET could have been considered, the extensive nature of the TAA meant a two-stage process was necessary regardless. The advantage of FET in this scenario is to provide a safe proximal landing zone for future TEVAR to treat residual disease in the descending aorta [4]. Furthermore, the Thoraflex Hybrid prosthesis has collapsible nitinol stents distally, permitting proximal clamping of the aorta if an open repair is planned distally [2].

Secondly, proximalization of the FET has been identified to provide several advantages in literature, particularly in relation to the FET indications of tearing distally [2]. This could also include proximal damage i.e. to the supra-aortic arch vessels or if there’s significant separation between these vessels which may focus decision-making towards a zone 0 approach in a patient with multiple co-morbidities. A retrospective study by Tsagakis *et al.* [5] defined proximal placement as zone 2 and below, and identified this to be a protective factor for 5-year survival. They recognize that facilitating debranching by using a zone 0 approach can minimize technical difficulty by providing a superficial view and reduced circulatory arrest times [5]. Similarly, zone 0 was found to promote aortic wall remodelling over CET [6], and in the context of type A aortic dissection, was found to reduce the false lumen size and increase the true lumen size. Further advantages have also been identified comparing zone 2 to zone 3 by Geragotellis *et al.* [7] such as reduced risk of recurrent laryngeal nerve injury due to reduced manipulation of the left subclavian artery.

However, it also important to consider the risks of a proximal approach. Akbulut *et al.* [8] identified that unresected aortic tissue, although the island technique wasn’t used here, has a risk of dilatation if there were future endoleaks. The incidence of stroke risk using zone 0 is also debatable. Yamamoto *et al.* [3] found a stroke incidence of 3.7% in their retrospective study of zone 0 arch repair for Type A Aortic Dissection and no spinal cord injury. On the other hand, Beckmann *et al.* [9] identified an elevated stroke risk of 19% in their population of FET both with and without root replacement which was non-significant. Differences in spinal cord ischaemia can possibly be attributed to stent placement occluding spinal arteries leading to malperfusion [10]. However, these studies were conducted in an emergency setting due to aortic dissection, unlike in our patient.

In conclusion, this innovative approach was possible due to the unique situation of contraindication to thoracotomy and acute bend of the distal arch combined with the extensive nature of the aneurysm. Zone 0 can be an effective mode of surgical treatment in an elective scenario of aneurysm repair using the FET with positive results.

## Data Availability

The authors confirm that the data supporting the findings of this study are available within the article and its supplementary materials.
